# Comparison of Reflectance Measurements Acquired with a Contact Probe and an Integration Sphere: Implications for the Spectral Properties of Vegetation at a Leaf Level

**DOI:** 10.3390/s16111801

**Published:** 2016-10-28

**Authors:** Markéta Potůčková, Lucie Červená, Lucie Kupková, Zuzana Lhotáková, Petr Lukeš, Jan Hanuš, Jan Novotný, Jana Albrechtová

**Affiliations:** 1 Department of Applied Geoinformatics and Cartography, Faculty of Science, Charles University in Prague, Albertov 6, 128 43 Prague 2, Czech Republic; lucie.cervena@natur.cuni.cz (L.Č.); lucie.kupkova@natur.cuni.cz (L.K.); 2 Department of Experimental Plant Biology, Faculty of Science, Charles University in Prague, Viničná 5, 128 44 Prague 2, Czech Republic; zuzana.lhotakova@natur.cuni.cz (Z.L.); jana.albrechtova@natur.cuni.cz (J.A.); 3 Global Change Research Institute, Academy of Sciences of the Czech Republic, v.v.i., Bělidla 986/4a, 603 00 Brno, Czech Republic; lukes.p@czechglobe.cz (P.L.); hanus.j@czechglobe.cz (J.H.); novotny.j@czechglobe.cz (J.N.)

**Keywords:** broadleaved leaf, broadleaved plants, conifers, contact probe, integration sphere, needle, spectroradiometer, spectroscopy

## Abstract

Laboratory spectroscopy in visible and infrared regions is an important tool for studies dealing with plant ecophysiology and early recognition of plant stress due to changing environmental conditions. Leaf optical properties are typically acquired with a spectroradiometer coupled with an integration sphere (IS) in a laboratory or with a contact probe (CP), which has the advantage of operating flexibility and the provision of repetitive in-situ reflectance measurements. Experiments comparing reflectance spectra measured with different devices and device settings are rarely reported in literature. Thus, in our study we focused on a comparison of spectra collected with two ISs on identical samples ranging from a Spectralon and coloured papers as reference standards to vegetation samples with broadleaved (*Nicotiana Rustica* L.) and coniferous (*Picea abies* L. Karst.) leaf types. First, statistical measures such as mean absolute difference, median of differences, standard deviation and paired-sample t-test were applied in order to evaluate differences between collected reflectance values. The possibility of linear transformation between spectra was also tested. Moreover, correlation between normalised differential indexes (NDI) derived for each device and all combinations of wavelengths between 450 nm and 1800 nm were assessed. Finally, relationships between laboratory measured leaf compounds (total chlorophyll, carotenoids and water content), NDI and selected spectral indices often used in remote sensing were studied. The results showed differences between spectra acquired with different devices. While differences were negligible in the case of the Spectralon and they were possible to be modelled with a linear transformation in the case of coloured papers, the spectra collected with the CP and the ISs differed significantly in the case of vegetation samples. Regarding the spectral indices calculated from the reflectance data collected with the three devices, their mean values were in the range of the corresponding standard deviations in the case of broadleaved leaf type. Larger differences in optical leaf properties of spruce needles collected with the CP and ISs are implicated from the different measurement procedure due to needle-like leaf where shoots with spatially oriented needles were measured with the CP and individual needles with the IS. The study shows that a direct comparison between the spectra collected with two devices is not advisable as spectrally dependent offsets may likely exist. We propose that the future studies shall focus on standardisation of measurement procedures so that open access spectral libraries could serve as a reliable input for modelling of optical properties on a leaf level.

## 1. Introduction

As many recent studies have proved, laboratory measurements of leaf optical properties in the visible and infrared regions are a valuable technique for understanding different plant physiological processes and stress detection [1,2,3,4,5], as well as photosynthesis efficiency evaluation, energy balance calculation, global terrestrial net primary productivity modelling [6,7,8,9] or vegetation stress detection [10,11,12,13,14]. Despite its wide applications, significant measurement uncertainties and knowledge gaps exist. These are related mainly to non-flat leaves, such as coniferous needles exhibiting a long and thin leaf type spatially oriented around a shoot [5,15,16,17,18].

Measurements of optical properties at the leaf level are typically acquired with a laboratory spectroradiometer coupled with an integrating sphere (IS). Within a sphere, light reflected or transmitted from a sample is integrated over a full hemisphere to yield measurements insensitive to sample anisotropic directional reflectance (transmittance) behaviour. This allows for repeatable measurements of vegetation samples.

Another mean of leaf optical properties’ acquisition includes contact measurements with a reflectance (contact) probe. A contact probe (CP) is a device mainly designed for contact measurements of solid raw materials such as minerals and grains, but also used for vegetation samples. Like an IS, a probe has its own light source (typically krypton halogen bulb) integrated within its body. A CP is retrofitted with a black, slip-on circular spacer that maintains a constant distance from the probe lens to the sample. In contrast to hemispherical measurements in an IS, a CP does not allow transmittance measurements. Also, some additional measurement ambiguities caused by multiple and multi-directional reflectance or possible damages of vegetation samples due to heat transferred from light source must be considered. However, use of a CP has some advantages like avoiding problems with stray light, operating flexibility and speed. Also, by its design, it allows for repetitive and non-destructive in-situ measurements of samples.

Though designed mainly for solid materials, CP measurements have also been successfully used for assessing metal stress in *Arabidopsis thaliana* plants [12], for estimating chlorophyll content in field crops [19] or detecting water stress in poplar at both the leaf and canopy levels [20]. Our research team used CP measurements within a framework of two projects. The first project focused on the assessment of mining-related impacts on selected tree species—Scots pine (*Pinus sylvestris* L.) and silver birch *(Betula pendula* Roth). We proved that measurements based on a CP (in our case an Analytical Spectral Devices, ASD Inc., Boulder, CO, USA contact probe) may provide valuable inputs for statistical modelling of vegetation parameters [4,21]. Laboratory spectroscopic data for the second project focused on the development and improvement of methods for monitoring of Norway spruce (*Picea abies* L. Karst.) health status in the Krušné hory Mts., Czech Republic [22,23]. Here, both an ASD CP and ASD IS were used.

In order to be able to cross-compare leaf optical properties measured by either contact probes or integrating spheres, one should be aware of the constancy among the measurements. A literature review revealed studies comparing reflectance values measured with different spectroradiometers [24,25,26], with a CP and a fore optic lens [27]; however, up to now no study on mutual comparison of a CP and an IS has been published. Particularly in the case of coniferous needles spatially arranged on a shoot, the methodology of measurement matters remarkably, possibly affecting the values obtained by different devices. There is a gap in experiments that would compare spectra measured on identical samples (standard samples or vegetation specimens or other materials e.g., soils, rocks) using a CP and an IS or experiments comparing measurements acquired by two or more different types of ISs. Confirmation of comparability of reflectance measured by a CP and an IS could bring simplification of field/laboratory vegetation optical properties measurements and their interpretation in some cases.

To address the above mentioned scientific issues, we designed an empirical laboratory experiment to compare spectra measured with different devices. Our main research questions were: (1) Are there differences between spectra collected with a CP and an IS? (2) Are the retrieved leaf biochemical properties obtained from spectral measurements performed with a CP and an IS yielding comparable results? (3) To what extent does the leaf type (broadleaved leaf and coniferous needles with their spatial arrangement on a shoot) affects spectral leaf properties obtained with a CP and an IS? We measured both reference standards (a Spectralon, coloured papers) and vegetation samples of both leaf types (broadleaved leaves of *N. rustica* and *P. abies* needles) using an ASD CP and two integrating spheres: An ASD IS (RTS-3ZCr2) and a Labsphere IS (RTC-060-SF) and we proposed a methodology for comparison of measurements obtained with different devices at three levels: Using raw spectra, derived vegetation indices and the quantitative retrieval of leaf-level biochemical parameters.

## 2. Materials and Methods

### 2.1. Materials

#### 2.1.1. Reference Materials

Although our methodology is focused on vegetation spectra, we also measured two types of artificial samples with stable optical properties—A Spectralon and a set of coloured papers. These represented stable reference materials, which do not change during the measurement process (e.g., due to a loss of water as in the case of some vegetation samples).

Reflectance was measured for a calibrated Zenith Lite^®^ Diffuse Reflectance Target (nominal reflectance of 95%, SphereOptics GmbH, Herrsching, Germany). The supplied calibration protocol was used as a reference. Next, reflectance spectra of nine different coloured papers were measured; the used paper-weight was 80 g/m^2^ for five colours: white, black, blue, light green and red, and 160 g/m^2^ for white, green, red and yellow colour. The impact of a substitution error (see Section 2.2) on measurements acquired with an IS was examined on the coloured paper samples.

#### 2.1.2. Vegetation Samples

**Figure 1:**
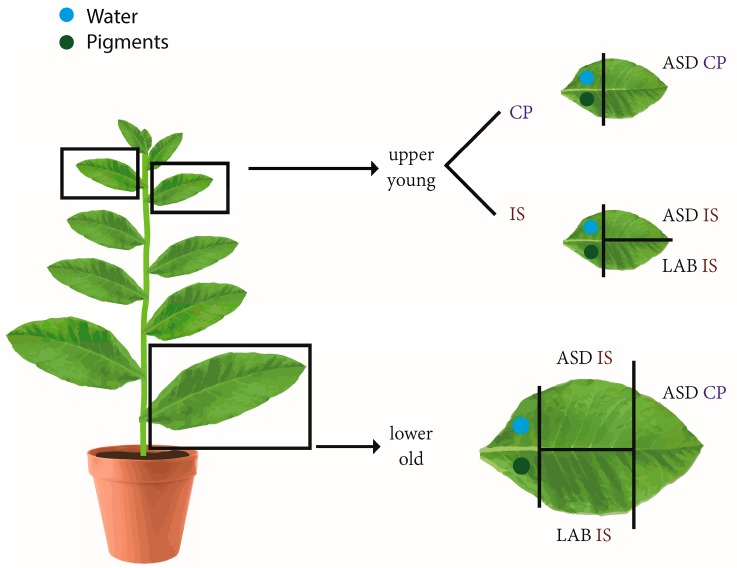
Design of the sampling and measurements in the tobacco experiment. CP—Contact probe, IS—Integrating sphere. ASD, LAB—Producers of the devices.

In our experiments, two types of plant samples were measured: tobacco leaves (*Nicotiana Rustica* Roth) as a representative of a ‘broadleaved’ leaf type, i.e., dorsiventral leaf, and Norway spruce (*Picea abies* L. Karst.) needles as a representative of a ‘coniferous’ leaf type:

*Tobacco leaves*: Tobacco plants (36 individuals) were grown in pots in a greenhouse for two months during early summer. Three leaves were measured per each plant. First, a mature leaf located in the lower part of the plant was divided into thirds and measured simultaneously using both the CP and the two ISs. Further, two younger leaves of subsequent insertion were cut; one was measured using the CP and the other one was divided in halves and measured simultaneously in the two IS. Finally, two leaf samples were used for the biochemical determination of photosynthetic pigments and water content. The design of the experiment is shown in Figure 1.

*Norway spruce needles*: The needles were collected in 2013 from mature even-aged forest stands in the Krušné hory mountains, Czech Republic [28]. In total, 55 trees were sampled—Reflectance spectra of the first two needle age classes from three vertical crown levels were measured. Next, the chlorophyll, carotenoids and water contents were biochemically estimated. After excluding outliers, 96 samples were used for the analysis. A detailed description of the dataset and evaluation of relations between biochemical and spectral measurements for Norway spruce needles can be found in [22,23].

**Figure 2:**
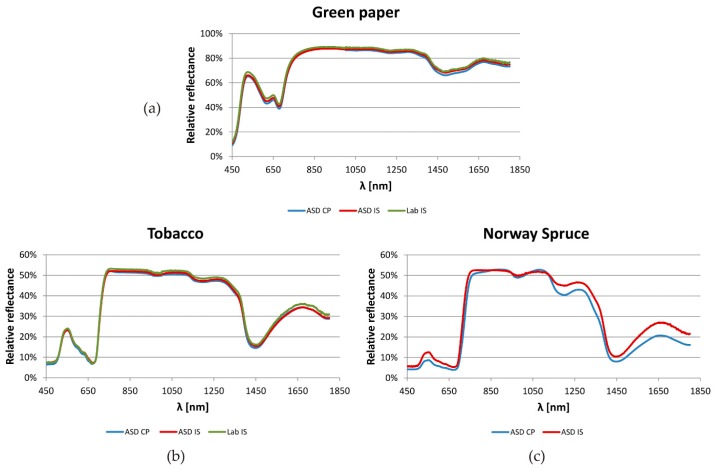
Examples of reflectance spectra of (**a**) coloured (green) paper and vegetation samples—(**b**) tobacco; (**c**) Norway spruce. CP—Contact probe, IS—Integrating sphere. ASD, LAB—Producers of the devices.

Examples of spectra of coloured papers, tobacco leaves and spruce needles collected with different devices are shown in Figure 2.

#### 2.1.3. Biochemical Measurements

Photosynthetic pigments were extracted using dimethylformamide according to [29] and determined specrophotometrically. Pigment contents were calculated applying equations published in [30] and expressed on dry weight basis (μg/cm^2^). Norway spruce needles were scanned before the pigment extraction. The ratio between the needle dry mass and needle projection area was calculated and used for conversion of amount of pigments to μg/cm^2^, the standard unit in vegetation spectroscopy and remote sensing [16]. In case of tobacco, the samples of constant area were cut from the leaf and amount of pigments was directly related to a leaf unit area (cm^2^).

The relative water content (RWC) was determined as the percentage of water in the fresh needles or leaves (the fraction of biomass weight decrease after drying). Fresh needles or leaves were weighed immediately after sampling, oven dried at 80 °C for 48 h and then weighed again.

### 2.2. Instruments

Spectral reflectance was measured in the range between 350 and 2500 nm using a FieldSpec 4 Wide-Res spectroradiometer (ASD Inc., Boulder, CO, USA). The ASD CP light source is a halogen bulb with colour temperature of approximately 2900 K whereas the ASD IS is supplied with a collimated tungsten light source [31]. According to the manufacturer’s protocol, subsequent reflectance and transmittance measurements of a sample requires changing the lamp position between two ports of the IS. It requires some time and it may introduce additional uncertainty due to the shift in light source position and intensity. Thus, in the next step we also tested the Labsphere integrating sphere (Lab IS, North Sutton, NH, USA), light source (KI-120 Koehler Illuminator with 120 W, 3200 K tungsten halogen lamp, Lab IS, North Sutton, NH, USA) of which is fixed during the measurements and only the sample is exchanged between the ports. However, due to the noise in data collected with the Lab IS, all calculations were performed only in the spectral interval from 450 to 1800 nm. Thus, Table 1 also characterises the ASD FieldSpec 4 Wide-Res spectroradiometer’s detectors just for this part of spectra: 512 elements silicon array for the visible and near infrared parts of the spectrum (350–1000 nm) and Graded Index InGaAs Photodiodes for the shortwave infrared part of the spectrum (SWIR1: 1000–1800 nm). Their wavelength accuracy is 0.05 nm and the final spectral curve is composed of bands 1 nm wide.

#### 2.2.1. CP Measurements

Samples were placed on a plate coated with black paint (albedo < 0.05) to minimize the reflection of radiation transmitted through the sample (see also [4,22]). The relative reflectance spectrum for each measurement was calculated as a ratio of the measured radiance of the sample to the radiance of 99% spectralon panel (white reference), according to Equation (1):

(1) $$R_{\mathrm{sample}\_\mathrm{rel}}=\frac{DN_{\mathrm{sample}}}{DN_{\mathrm{WR}}}$$

where *R*_sample_rel_ is the relative reflectance of the sample, *DN*_sample_ is the measured reflected radiation from the sample (in DN values), *DN*_WR_ is the measured reflected radiation of the non-calibrated 99% Spectralon (white reference; the calibration protocol was not available for this Spectralon panel).

Five measurements were taken on different parts of a sample. The sample-specific value was calculated as a median of these five individual measurements. In case of tobacco, single leaf was selected for the measurements, whereas for Norway spruce, needles of the same age class (same shoot) were arranged in a stack still keeping spatial orientation on a shoot (upper part of the needles oriented upwards), see also [27]. Different scan averaging was applied for CP and IS measurements to avoid overheating of samples measured by the CP (Table 2).

#### 2.2.2. IS Measurements

All samples were measured according to the manufacturer’s protocol [31]. Because only reflectance measurements could be compared between the IS and CP, transmittance was not in focus in our study and is not discussed in the present paper. The scan averaging for all measurements was set up to 100 (Table 2) to improve the signal to noise ratio.

**Figure 3:**
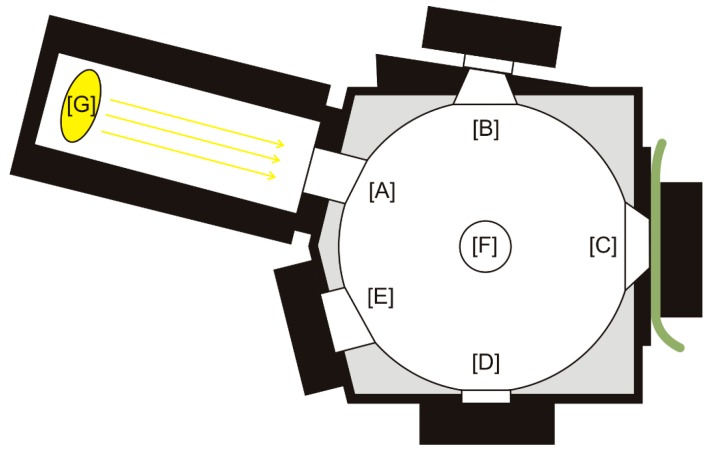
Schema of the ASD integration sphere and its ports: [A] Reflectance input; [B] Reflectance comparison; [C] Reflectance sample; [D] Transmission input; [E] Specular Exclusion Light Trap; [F] Fiber Adapter Port; [G] Collimated Light Source Assembly. The picture shows the setup of sample‘s reflectance measurement. For the white reference measurement content of the ports [B] and [C] are changed as described in the Table 3.

For narrow leaf samples (i.e., spruce needles) so called gap-fraction correction was further applied (see e.g., [5,15,18]). To assess the impact of the substitution error (SE, the error caused by the difference in the total energy collected with the optical cable when the reference and sample are placed in the port) samples were measured in two configurations (see Table 3); Figure 3 shows the scheme of the ports of the ASD IS.

The relative reflectance spectra of the colour papers and tobacco leaves were computed based on the abovementioned standard Equation (1) described in the manufacturer’s manual [31]. The stray light correction was not taken into account as the errors are negligible (it yields maximum of 0.01% of measured sample reflectance). In the case of the Spectralon, the absolute reflectance values were calculated as:

(2) $$R_{\mathrm{sample}\_\mathrm{abs}}=\frac{DN_{\mathrm{sample}}}{DN_{\mathrm{WR}}}\times R_{\mathrm{WR}}=R_{\mathrm{sample}\_\mathrm{rel}}R_{\mathrm{WR}}$$

where *R*_sample_abs_ is the absolute reflectance of the sample, *DN*_sample_ is the measured reflected radiation from the sample (in *DN* values), *DN*_WR_ is the measured reflected radiation from the 99% Spectralon (white reference) and *R*_WR_ is the calibrated reflectance value of the 99% Spectralon.

Due to their size, measurements of Norway spruce needles require more complex approach, which includes the gap-fraction correction of samples placed in a special sample-holder [33], revised by [15], summarized and extended in [5,18]. The gap-fraction is typically retrieved from the scans of sample-holders. The relative reflectance spectra of the needles are then derived from the measured radiance and the gap-fraction according to the Equation (3) [5,15]:

(3) $$R_{\mathrm{needle}}=\frac{\frac{\left(DN_{\mathrm{sample}}-DN_{\mathrm{straylight}}\right)}{DN_{\mathrm{WR}}-DN_{\mathrm{straylight}}}}{1-GF}$$

where *R*_needle_ is the reflectance of individual needles, *DN*_sample_ is the measured reflected radiation from the sample, i.e., needles + gaps in *DN* values, *DN*_straylight_ is the measured stray light radiation in *DN* values, *DN*_WR_ is the measured reflected radiation from the calibrated 99% Spectralon (white reference) in *DN* values, *GF* is the gap-fraction.

Measurements with all devices were carried out in a spectroscopic laboratory. In the case of the Spectralon, colour papers and spruce needles measurements, the ASD IS and ASD CP were subsequently connected to one ASD FieldSpec 4 Wide-Res spectroradiometer; another spectroradiometer of the same type was used for the Lab IS measurements. Tobacco samples were simultaneously measured with all three devices connected to three ASD FieldSpec 4 Wide-Res spectroradiometers.

### 2.3. Methodology of Spectra Comparison

The most reliable spectra comparison would be based on the absolute reflectance values calculated according to Equation (2). This approach was used in the case of the first test with the calibrated the 95% Zenith Lite^®^ Diffuse Reflectance Target, further called the 95% Zenith Lite^®^ Sepectralon calibrated reference. CP measurements were carried out with a non-calibrated 99% Spectralon. Equation (1) was then used for calculating reflectance values, which were relative to the 99% Spectralon. This approach widely applied in the field campaigns was utilized in the case of colour papers as well as vegetation experiments mostly for practical reasons—The diameter of an internal ASD IS 99% Calibrated Reference Standard just covers the ASD CP field of view and is therefore a potential source of errors. Using the 95% Zenith Lite^®^ Spectralon as a white reference is complicated in the ASD IS measurements because the spectralon is relatively big and difficult to be held in the port. Moreover, it does not fit to the above mentioned internal 99% standard coating the inside of the IS.

The rationale for spectra comparison is based on the following idea. If we assume a theoretical case of equal absolute reflectance values derived from a CP and an IS, their difference can be expressed as:

(4) $$R_{\mathrm{CP}\_\mathrm{abs}}-R_{\mathrm{IS}\_\mathrm{abs}}=R_{\mathrm{CP}\_\mathrm{rel}}R_{\mathrm{WR}\_\mathrm{CP}}-R_{\mathrm{IS}\_\mathrm{rel}}R_{\mathrm{WR}\_\mathrm{IS}}=0$$

where *R*_rel_ and *R*_abs_ are the absolute and relative reflectance values calculated according the Equations (1) and (2), respectively, and *R*_WR_ is the calibrated reflectance of the Spectralons used for the CP and IS measurements.

Thus, the relation between the relative measurements can be modelled with a linear function, multiplicative term of which corresponds to the unknown ratio *C*_WR_ between the reflectance values of the used Spectralons:

(5) $$R_{\mathrm{CP}\_\mathrm{rel}}=R_{\mathrm{IS}\_\mathrm{rel}}\frac{R_{\mathrm{WR}\_\mathrm{IS}}}{R_{\mathrm{WR}\_\mathrm{CP}}}=R_{\mathrm{IS}\_\mathrm{rel}}C_{\mathrm{WR}}$$

The test based on the calibrated Spectralon revealed an offset *C*_Oabs_ = *R*_CP_abs_ − *R*_IS_abs_ between the reflectance values derived from CP and IS measurements. Equation (5) was therefore extended to a full linear model described with Equation (6):

(6) $$R_{\mathrm{CP}\_\mathrm{rel}}=R_{\mathrm{IS}\_\mathrm{rel}}\frac{R_{\mathrm{WR}\_\mathrm{IS}}}{R_{\mathrm{WR}\_\mathrm{CP}}}+\frac{C_{O\_\mathrm{abs}}}{R_{\mathrm{WR}\_\mathrm{CP}}}C_{\mathrm{Oabs}}=R_{\mathrm{IS}\_\mathrm{rel}}C_{\mathrm{WR}}+C_{O}$$

where *C*_O_ is the unknown offset of absolute values *C*_Oabs_ divided by *R*_WR_CP_. The coefficients *C*_WR_ and *C*_O_ are spectrally dependent.

The measurements of colour papers, tobacco leaves and Norway spruce needles collected with different devices were first compared using selected statistical quantities. The mean absolute difference was applied as a measure of a mean magnitude of differences in reflectance. The median of differences quantifies a systematic shift between the compared spectra. The standard deviation was added to describe the variability of the differences around the mean value. First, the standard deviation was calculated from all samples of the same type of material acquired by one device for each wavelength between 450 and 1800 nm. To quantify the differences among spectra using a single quantity, a mean of standard deviations was then computed. Since the measurements were carried on the same samples with all devices, the similarity between the spectra was also evaluated on each wavelength and all combinations of devices by means of the paired-sample t-test with the level of significance *α* = 0.05. Furthermore, it was possible to estimate the coefficients of linear relation between the *R*_CP_rel_ and *R*_IS_rel_ values for each studied wavelength according to Equation (6). The similarity between the transformed spectral curves obtained from different devices was again evaluated based on the mean absolute difference, the mean standard deviation and the paired-sample t-test.

Normalized differential indexes (NDI) are commonly used when the relation between the spectral response of vegetation and its biochemical parameters are sought. Equation (7) represents a general expression of the normalised differential vegetation index calculated for reflectance values R on the wavelengths λ_1_ and λ_2_:

(7) $$NDI_{\lambda_{12}}=\frac{R_{\lambda_{2}}-R_{\lambda_{1}}}{R_{\lambda_{2}}+R_{\lambda_{1}}}$$

The NDI has a value from the interval <−1; 1>. It slightly differs if the relative or absolute reflectance is used for calculation. Due to the lack of calibrated spectralon for the ASD CP, the NDI were calculated from the relative reflectance values in our experiments. Based on the NDI values of samples measured either by the CP and the IS, the differences and correlation in NDI values were evaluated for colour papers and plant samples. In addition, the correlation of NDI with selected leaf compounds (total chlorophyll, carotenoids and water content) obtained from all three devices was calculated to demonstrate its applicability for quantitative remote sensing of vegetation.

Finally, we assessed the relationship between spectral indices often used in quantitative remote sensing of vegetation and leaf compounds. A linear regression and a calculation of a coefficient of determination R^2^ for all used devices and about fifty indices, summarized for chlorophyll in [34,35], for carotenoids in [36,37] and for water in [38] (pp. 232–233), were performed. Based on the results, four indices listed in Table 4 are presented further in this study.

## 3. Results and Discussion

First, spectral measurements collected with three device settings, ASD CP, ASD IS and Lab IS were directly compared and evaluated. Then the relationship between spectral measurements and leaf compounds was assessed. The reflectance values are presented as a reflectance factor (i.e., %). To avoid further confusion, a reader should note that the differences and standard deviations of reflectance are also given in %; however they describe absolute, between-sample differences and not relative values.

### 3.1. Spectra Comparison

#### 3.1.1. Calibrated Reference

First, reflectance of the 95% Zenith Lite^®^ calibrated reference was measured with the ASD IS and ASD CP. It was used both as a white reference and the sample, i.e., the ratio between the collected spectra—Relative reflectance, see Equation (1), should be close to 1 and possible deviations reflect the noise in the signal. Two measurements were carried out with both device settings.

**Figure 4:**
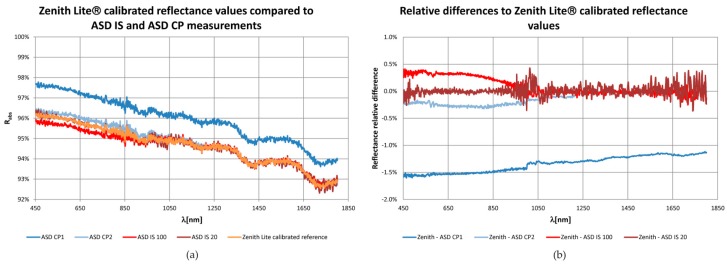
Comparison of the 95% Zenith Lite^®^ Spectralon calibrated reflectance values (*R*_ZL_) with reflectance determined by means of ASD IS and ASD CP measurements. (**a**) Original reflectance curves; (**b**) The relative differences calculated as (*R*_ZL_ − *R*_ASD IS/CP_)/*R*_ZL_.

In the case of the ASD IS, 20 and 100 of scans per measurement were used. The absolute reflectance measurements, according to the Equation (2), from the ASD IS showed a high correspondence to the calibrated reflectance values of the 95% Zenith Lite^®^ calibrated reference regardless the number of averaged scans. The 99% quantile of absolute differences between the collected and reference reflectance values in the spectral interval from 450 nm to 1800 nm equalled to 0.3% and 0.4% for 20 and 100 scans, respectively. The higher number of scans yields better SNR [45] what was also confirmed in our study (Figure 4). Based on this result, averaging of 100 scans was used for further measurements.

As shown in Figure 4, the shape of the reflectance curves acquired with the ASD CP was also similar to the calibrated standard, nevertheless one of them (called as ASD CP1 in Figure 4) revealed a systematic shift of 1.3%. Both measurements were performed under the same conditions and close in time. A closer look at raw data revealed that the CP optics had not been in a full contact with the Spectralon when measuring the white reference. The recorded *R_WR_* values were therefore lower what caused a higher *R*_abs_. The second measurement (ASD CP2) fits the 95% Zenith Lite^®^ calibrated reference. The 99% quantile of absolute differences in reflectance equals to 0.3% what is comparable to the ASD IS.

Relative differences (Figure 4) emphasized discrepancies between both devices, especially in the spectral interval between 450 and 1000 nm, where the CP slightly overestimated while the IS slightly underestimated the calibrated reflectance values. Also, a spectral shift between two detectors at VNIR and SWIR1 at 1000 nm was present in the data. The paired-sample t-test revealed significant differences between the spectra at α = 0.05.

#### 3.1.2. Coloured Papers

Due to the lack of a calibrated Spectralon for the CP measurements, only relative spectra of papers measured with the ASD CP, ASD IS and Lab IS were compared. The influence of the substitution error (SE) correction on spectra measured with the IS was also studied.

First, differences between original spectra were evaluated. As Table 5 shows, the best agreement between the relative reflectance values was achieved between ASD IS and Lab IS when the correction for the SE was applied. The reflectance values of the Lab IS were about 1.3% higher and the standard deviation was 0.5%. When comparing the IS and CP measurements, the correction of the SE changed the offset of the spectra while it had almost no influence on the standard deviation. Due to the offset between the spectra, the paired-sample t-test revealed significant differences between compared devices for all wavelengths with the only exception of ASD CP and ASD IS spectra without a SE correction. After including the mean difference between devices calculated for each wavelength, all combinations of spectra passed the paired-sample t-test on all wavelengths, i.e., no significant differences were observed.

In the next step, linear regression parameters according to Equation (6) between reflectance values of colour papers were calculated for each combination of devices and wavelengths. After applying the linear model, the offset between the spectra was eliminated, the absolute difference decreased in most cases and the standard deviation was practically preserved (see Table 5). It is worth mentioning that after the regression, the mean absolute residual between the measurements in the IS—when the SE was corrected—was on the level of results when ASD IS reflectance was compared to the 95% Zenith Lite^®^ calibrated reference. The linear regression lowered differences in a comparison of CP and IS with and without the SE correction; the mean absolute residuals of 0.9% and standard deviation of 1.1% were achieved.

**Figure 5:**
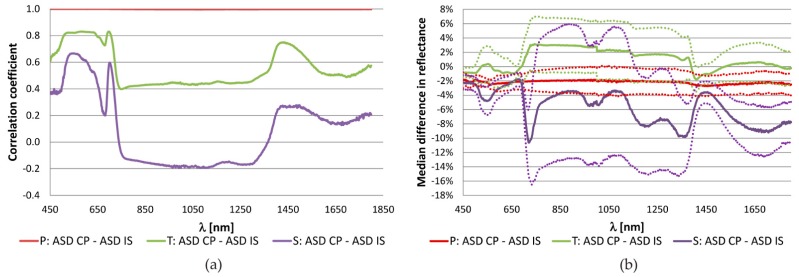
Spectral dependence of (**a**) the correlation coefficient and (**b**) the mean difference in reflectance obtained from the comparison of available samples measured with the ASD contact probe (CP) and ASD integration sphere (IS) for each wavelength in the spectral interval from 450 nm to 1800 nm. P—Coloured papers (9 samples), T—Tobacco leaves (72 samples), S—Norway spruce (96 samples). The dot lines around the median difference in reflectance show the interval of ± standard deviation.

An example demonstrating the spectral dependence of three selected statistical measures evaluating relative reflectance values derived for the same samples using the ASD CP and ASD IS is shown in Figure 5. In contrast to measured leaves, the differences are more spectrally invariant in the case of colour papers what could be caused by a lower variability in colour paper reflectance especially in the NIR part of the spectrum.

#### 3.1.3. Vegetation Samples

The spectra comparison of tobacco leaves and Norway spruce samples was carried out in the same way as in the case of the colour papers. The application of the SE correction on measurements of tobacco samples brought changes in the offset of the spectra and did not significantly alter the other statistical values (see Table 6). As the correction is fully supported from the theoretical point of view, only measurements with applied correction for SE were used in all the following experiments.

The comparison of the original tobacco spectra revealed similar results for all three devices; the smallest offset was observed between the ASD CP and ASD IS measurements (see Table 6). The paired-sample t-test proved significant differences between all measured spectra. Subtracting the offset between the spectra calculated for each wavelength from one of the compared measurements was sufficient to pass the paired-sample t-test for all combinations of devices with *p*-values close to 1.

Table 6 also shows that the application of the linear regression decreased the absolute differences in spectral values with a factor of 1.5 to 2 and in the case of comparison of the CP and ISs it also lowered the standard deviation. In comparison to the coloured papers, the higher values of statistical measures were mainly caused by a higher variability of optical properties of the samples and their slightly different insertion on a plant as it was demonstrated in Figure 1. The correlation coefficient between spectral measurements of 72 samples in each wavelength was lower (*r*_T_mean_ = 0.558) than in the case of coloured papers (*r*_P_mean_ = 0.998), see Figure 5.

Castro-Esau et al. [24] compared reflectance spectra of three leaf plants (*Cafea Arabica*, *Lantana camara*, *Eriobotrya japonica*) collected with three different spectroradiometers by means of D metric that corresponds to Root Mean Square Error (RMSE). Using the same IS but different spectrometers they received the D value about 0.5%. Using different spectrometers and different devices for data collection (optical cable, pistol grip), the D values increased to 2%–6% which is the range of RMSE in Table 6.

Norway spruce samples were measured with the ASD CP and ASD IS (including the SE correction). The statistical values describing differences between the two devices were in most cases doubled in comparison to the tobacco samples (Table 6). The correlation coefficient between the two measurements was low (ranging from −0.2 to 0.2) in the large spectral interval from 750 nm to 1400 nm as shown in Figure 5a. The variability among the samples was the highest of all tested materials as indicated by the standard deviation values both in Figure 5 and Table 6. The t-test applied on the original data proved significant differences between the spectra on all used wavelengths. Significant differences did not exist anymore after the linear transformation of CP spectra. The larger discrepancies in comparison to other tested materials were mainly influenced by a different measurement procedure. While only a single layer of needles was measured in the IS, a compact layer of needles on shoots causing multiple, multidirectional reflectance between the layers (needles) but also a loss of signal caused a difference in radiances recorded with the CP. The median of differences was negative what implicated the higher reflectance values measured with the ASD IS. This result was in correspondence with [46] dealing with an upscaling model from needle to shoot spectra. The application of such model would require estimation of a shoot structural parameter which was not performed in the scope of this study. The results are therefore biased by omitting this correction step.

#### 3.1.4. Normalized Differential Index

In the next step, the normalised differential index was calculated for all measured samples, devices, device settings and combinations of wavelengths in the interval between 450 nm and 1800 nm. The summary statistics of differences in NDI between the devices is shown in Table 7. The differences in NDI were at the level of 0.01 in the case of the coloured papers. Due to a higher variability and a higher number of samples, the differences in the plant material were higher, with a factor of 2–3 in the case of tobacco and with the factor of 6 and 3 for the mean absolute difference and standard deviation, respectively, in the case of Norway spruce. The linear transformation of spectra brought improvements for most measurements.

**Figure 6:**
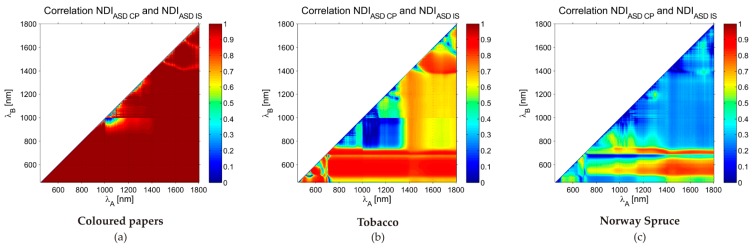
Spectral dependence of correlation coefficient (its absolute value) between normalized differential index (NDI) values derived from relative reflectance measured with the ASD CP and ASD IS for (**a**) coloured papers; (**b**) tobacco leaves; (**c**) Norway spruce needles. CP—Contact probe, IS—Integration sphere, ASD, Lab—Producers of the measurement devices.

The spectral dependency of differences in the NDI is demonstrated in Figure 6. It shows examples of the correlation coefficient between the NDI values calculated for different materials from all samples and all combinations of wavelengths for the ASD CP and ASD IS. It generally confirms the increasing discrepancy between the spectra obtained by different instruments and device settings when moving from relatively homogeneous materials (i.e., coloured papers and tobacco leaves) to more complicated measurements of Norway spruce needles. The graphical results are, thus, in correspondence with statistics summarized in Table 7. Figure 6 also follows the results shown in Figure 5a. The high correlation in original spectra emanates high correlation in NDI and vice versa. The drop of correlation values around 1000 nm is caused by the spectral shift between the spectroradiometer’s sensors.

### 3.2. Retrieval of Leaf Biochemical Properties

The NDI values were also correlated with the content of photosynthetic pigments and water content determined with the laboratory analyses as described in Section 2.2. The summary of the mean contents of chlorophyll, carotenoids and water is given in Table 8.

**Figure 7:**
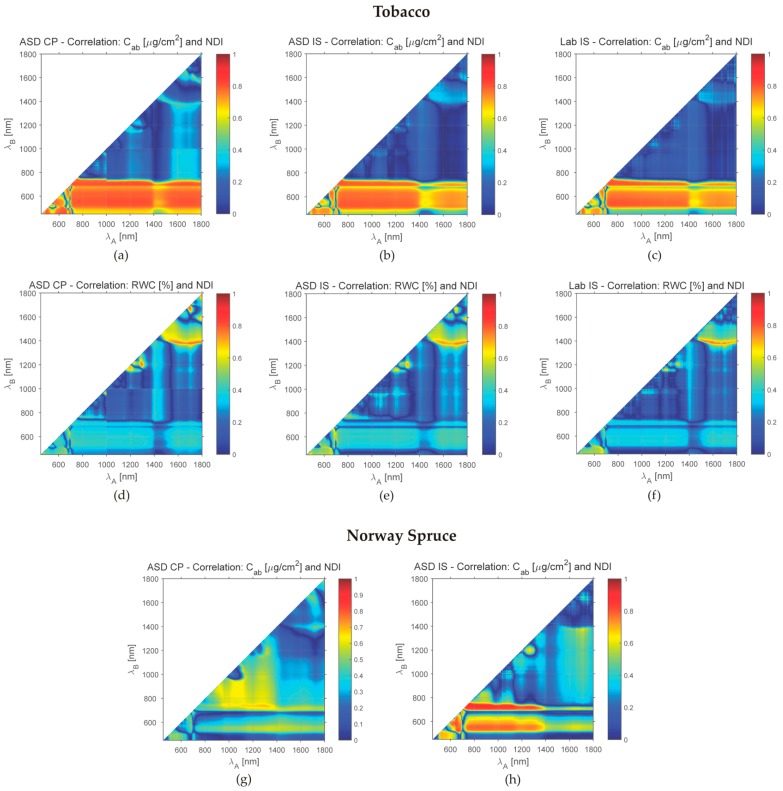

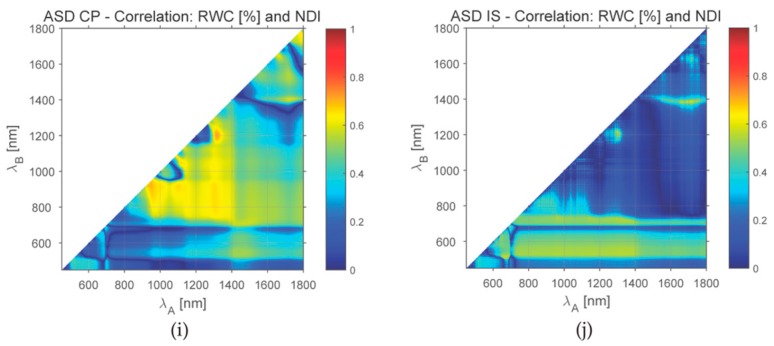
Absolute values of the correlation coefficient between leaf compounds (total chlorophyll—Cab and relative water content—RWC) and NDI (normalised differential index) derived for (**a**–**f)** tobacco samples and (**g**–**j**) Norway spruce and the used devices. CP—Contact probe, IS—Integration sphere, ASD, Lab—Producers of the measurement devices.

The correlation coefficients between all three leaf compounds and NDI derived for tobacco and Norway spruce samples from all available reflectance measurement were calculated. Figure 7 depicts examples of correlation between chlorophyll and NDI, RWC and NDI (the relations of NDI to carotenoids are not presented; contents of carotenoids and chlorophyll are highly correlated and the diagrams show almost identical patterns). In spite of differences in the correlation coefficient values, the diagrams show similar trends for all three devices in the case of tobacco. The NDI derived from the ASD IS measurements of Norway spruce needles have stronger correlation to chlorophyll in the combination of spectral ranges 700–1400 nm and 550–750 nm in comparison to the NDI derived from ASD CP. The correlation to water content is in general low for both types of vegetation samples with a narrow peak region at 1500–1800 nm and around 1400 nm (water absorption band). A detailed study of local maxima in Figure 7 could provide a base for proposing spectral indices sensitive to leaf compounds. Our study did not aim at such analysis; higher variability in samples would be necessary for derivation and validation of such indices.

Finally, the relationship between the leaf compounds and corresponding spectral indices derived from different devices was evaluated. Table 9 gives an overview of indices that revealed the highest values of the coefficient of determination (R^2^). With the exception of the chlorophyll content, the R^2^ were too small to prove a significant relationship between the leaf compounds and corresponding indices.

The main reason is a low variability in carotenoids and water content in the dataset (see Table 8) despite our efforts to introduce differences in physiological status of sampled plants (e.g., different regimes in watering and using both mature and young leaves in the case of tobacco). This was probably caused by the fact that tobacco plants are not well structurally adapted to a water loss, e.g., by thick cuticle or presence of hairs. However, tobacco is a standard model plant species, reliably and fast growing in greenhouse conditions.

The performed t-tests showed that majority of the fifty calculated indices differed significantly between the tested devices. Among indices presented in Table 9, the null hypothesis about the non-significant differences between the indices derived from tobacco samples was confirmed only for following combinations of devices: OSAVI 2—ASD IS & Lab IS; R_NIR_CRI_550_—ASD CP & ASD IS, ASD CP & LAB IS.

As Table 9 also shows, the mean values of indices among devices differed within the standard deviation in the case of tobacco. Looking at coniferous samples, the mean values of indices obtained with the ASD IS and the ASD CP differed with a factor of 1.5 to 2. The reason for this is obviously in the measurement procedure connected with leaf type as described in Section 3.1.3.

## 4. Conclusions

The objective of our study was to test whether the spectral measurements collected with a CP and an IS yield comparable results. The main motivation of this study arose from our practical experience with the CP measurements being both convenient for field work and also less time consuming than the IS measurements. The comparison of spectra collected with two ISs was another objective as the data exchange between research groups is frequent and comparability and quality are then important issues. It must be stressed out that the present conclusions are based on the datasets where the relative reflectance values were used due to a lack of a calibrated Spectralon used for the CP measurements. It is assumed that using of absolute reflectance values could result in further improvement.

The calibrated reflectance values of the 95% Zenith Lite^®^ calibrated reference and absolute reflectance measured with the ASD IS and ASD CP differed less than 0.5% (in relative units) in the best cases, which is on the level of the instrument performance [47]. In order to achieve such a result, attention has to be paid to both the measurement itself in order to avoid possible measurement errors (such as a loose contact between the contact probe and the sample) and rigorous data post-processing (e.g., excluding outliers from the measurements). The effect of the stray light correction was small (less than 0.01%) and, thus, not significant for our experiments.

The experiment with the coloured papers revealed higher discrepancies between spectral reflectance values obtained from different devices and device settings in comparison to the Spectralon. It showed that differences exist even when two ISs of a different manufacturer are compared. The main conclusions are that (i) the best agreement was achieved between measurements from ASD IS and Lab IS when the correction for substitution error was applied; (ii) if the samples measured with the same devices exist, they can be used for derivation of parameters of a linear transformation (at each wavelength) that can bring the compared spectra to a better agreement. Based on following tests with two different leaf types—tobacco and Norway spruce—this transformation gave better results for broadleaved than for coniferous vegetation.

The last objective of the study was to show to what extent does the leaf type (broadleaved leaf and coniferous needles with their spatial arrangement on a shoot) affect spectral leaf properties obtained by a CP and an IS. The study revealed that in the case of broadleaved leaf type, the differences in using the IS and CP are smaller than in the case of coniferous needles. Coniferous needles have a thick cuticle and rhomboid shape on cross section. In addition, needles are attached to a shoot under different angles and this spatial architecture, which causes multidirectional reflectance, may affect measurements with a CP since needles are kept on a shoot during these measurements. In contrast to measurements with an IS where single, detached needles are laid parallel on a tray of a sample holder and, thus, shoot architecture does not interfere with measurements. In future studies, models for shoot spectral properties based on needle spectral measurements shall be applied and evaluated. As a broadleaved plant species in our case we used tobacco with a leaf type without a specific anatomic adaptation of epidermis. It may happen that broadleaved leaf types with pronounced epidermal structure corresponding to more xeropmorphic leaf adaptations could exhibit more differences, though.

The relation between NDI derived from spectra collected by different devices followed the observation that in spite of our effort to have an identical sample per one set of measurements, some individual variability had been present (increasing from papers, through tobacco to spruce needles). It caused higher differences in corresponding spectra and subsequently also higher differences in the derived NDI. The data collected with the ASD FieldSpec 4 Wide-Res spectroradiometer were sampled with the interval of 1 nm. The noise in data could influence the calculated NDI. Therefore smoothing original spectra with a suitable filter, e.g., a Savitzky–Golay filter [48,49] could improve the results. The conclusions about NDI are valid also for specific vegetation indices used in quantitative remote sensing for modelling of relation between spectral observations and the content of biochemical or biophysical compounds in vegetation.

The present study contributes to an effort in using a more coherent approach of leaf optical property acquisition, which is essential for using such data in standardization when upscaling to a canopy level. Leaf optical properties measured at leaf level can directly enter to radiative transfer models at canopy level and contribute significantly to their parametrization and further to simulation of imaging spectrometer data [50]. The study shows that a direct comparison between the spectra collected with two devices is not advisable as spectrally dependent offsets may likely exist as we demonstrated for the compared devices and device settings. They are caused by the construction of the devices, light sources used, measurement procedures and properties of the measured material, e.g., leaf type and structure. As an implication of our results for remote sensing vegetation studies we can recommend to be very careful with comparisons of laboratory spectral measurements conducted with different devices and under different conditions. The experiments should be documented in detail in order to be repeatable and reproducible, and the same devices should be used in the case of mutual comparison. When using two or more devices a definition of a linear model transforming a spectrum of one device to another one is a solution that decreases the differences. In order to find parameters of such a model, a subset of the samples has to be measured with both devices which in principle corresponds to the approach of introducing an internal standard suggested for soil spectral measurements [26]. Elaboration on procedures that enable to work with spectra on a leaf level acquired with different devices would open a possibility of using various open access spectral databases and spectral libraries as a robust data source for spectroscopy modelling on a leaf level.

## Figures and Tables

**Table 1:** ASD FieldSpec 4 Wide-Res spectroradiometer specification [32].

Parameter	VNIR (350–1000 nm)	SWIR1 (1001–1800 nm)
Material of the detector	Silicone	InGaAs, TE Cooled
Spectral resolution	3 nm (at 700 nm)	30 nm (at 1400 nm)
Noise Equivalent Radiance (NEdL, W/cm^2^/nm/sr)	1.0 × 10^−9^ (at 700 nm)	1.5 × 10^−9^ (at 1400 nm)
Stray light	0.02%	0.01%
Maximum radiance	2× Solar	10× Solar

**Table 2:** The measured material and methods. IS—Integration sphere, CP—Contact probe, ASD, Lab—Device manufacturers.

Material	Number of Samples	Instrument	Scan Averaging	Methods of Measurement	
Spectralon	1	ASD CP	20	Absolute reflectance (comparison with the calibration data)	
		ASD IS	100 (20)	Absolute reflectance (comparison with the calibration data)	
Colour papers	9	ASD CP	20	Five measurements—Median spectrum	Relative reflectance
		ASD IS	100	Substitution error vs. no substitution error	
		Lab IS	100	Substitution error vs. no substitution error	
Tobacco leaves	72	ASD CP	20	Five measurements—Median spectrum	
		ASD IS	100	No substitution error	
		Lab IS	100	No substitution error	
Norway spruce needles	96	ASD CP	50	Five measurements—Median spectrum	
		ASD IS	100	Substitution error	

**Table 3:** Configurations of ASD integrating sphere. SE—Substitution error, see the text.

Method	Measured Quantity	Sphere Ports Configuration				
		Port A	Port B	Port C	Port D	Port E
1—Does not correct for SE	*R*_WR_	L	W (uncal)	W (cal)	P	P
	*R*_sample_	L	W (uncal)	S	P	P
2—Corrects for SE	*R*_WR_	L	S*	W (cal)*	P	P
	*R*_sample_	L	W (uncal)*	S*	P	P

SE—Substitution error, see the text, *R*_WR_—Reflectance of the white reference, *R*_sample_—Reflectance of the sample, L—Integrating sphere external light source; W—White reference (uncal—Uncalibrated Zenith standard, cal—Calibrated Zenith standard), S—Sample, P—White plug, *—For Norway Spruce needles measurements the sample holder was added [18].

**Table 4:** Evaluated spectral indices. R is reflectance on a given wavelength.

Index Name	Formula	Reference
Optimized Soil-adjusted Vegetation Index 2	OSAVI2 = (1 + 0.16) * (R_750_ − R_705_)/(R_750_ + R_705_ + 0.16)	[39]
Carotenoid Vegetation Index (R_NIR_ * CRI_550_)	R_NIR_CRI_550_ = ((1/R_510_ − 1/R_550_) * R_770_)	[40,41]
Moisture Stress Index	MSI = R_1600_/R_820_	[42]
Transformed Chlorophyll Absorption Ratio Index	TCARI = 3 * [(R_700_ − R_670_) − 0.2 * (R_700_ − R_550_) * R_700_/R_670_)]	[43]
Carotenoid Vegetation Index	CRI_700_ = 1/R_515_ − 1/R_700_	[40]
Normalise Differential Water Index	NDWI = (R_857_ − R_1241_)/( R_857_ + R_1241_)	[44]

**Table 5:** Comparison of differences in coloured papers relative reflectance [%] measurements by three devices before (Mean absolute difference, Median of differences, Mean standard deviation, Root Mean Square Error—RMSE) and after linear regression (Lin. reg. mean absolute residual, Lin. reg. mean st. d.—Linear regression mean standard deviation). Measurements that were not corrected for the substitution error are in parenthesis. CP—Contact probe, IS—Integration sphere, ASD, Lab—Producers of the measurement devices.

Differences in Relative Reflectance [%]	Mean Absolute Difference	Median of Differences	Standard Deviation	RMSE	Lin. reg. Mean Absolute Residual	Lin. Reg. Mean st. d.
ASD CP–ASD IS	2.4 (1.3)	−2.2 (0.4)	1.5 (1.7)	2.7 (1.8)	1.0 (1.1)	1.2 (1.4)
ASD CP–Lab IS	3.6 (1.8)	−3.6 (−1.5)	1.4 (1.2)	3.9 (2.0)	0.9 (0.7)	1.1 (0.9)
ASD IS–Lab IS	1.4 (2.3)	−1.3 (−2.3)	0.5 (0.7)	1.5 (2.4)	0.3 (0.6)	0.4 (0.7)

**Table 6:** Comparison of differences in tobacco leaves (T) and Norway spruce (S) relative reflectance [%] measurements before (Mean absolute difference, Median of differences, Mean standard deviation, Root Mean Square Error - RMSE) and after linear regression (Lin. reg. mean absolute residual, Lin. reg. mean standard deviation—st. d.). Measurements that were not corrected for the substitution error are in parenthesis. CP—Contact probe, IS—Integration sphere, ASD, Lab—Producers of the measurement devices.

Differences in Relative Reflectance [%]	Mean Absolute Difference	Median of Differences	Standard Deviation	RMSE	Lin. Reg. Mean Absolute Residual	Lin. Reg. Mean st. d.
T: ASD CP–ASD IS	2.5 (5.0)	0.7 (4.6)	2.9 (2.9)	3.1 (5.4)	1.5	2.0
T: ASD CP–Lab IS	2.4 (2.5)	−1.3 (0.6)	2.7 (2.8)	3.4 (3.1)	1.5	1.9
T: ASD IS SE–Lab IS	2.5 (4.2)	−2.0 (−4.1)	1.8 (1.8)	3.0 (4.5)	1.3	1.7
S: ASD CP–ASD IS	6.8	-5.2	5.0	7.9	2.8	3.4

**Table 7:** Mean absolute difference (MAD) and mean of standard deviations (MStD) in normalized differential index (NDI) differences calculated for all available samples and all combinations of wavelengths in the interval 450 nm–1800 nm. CP—Contact probe, IS—Integration sphere, ASD, Lab—Producers of the measurement devices.

**NDI Differences: Coloured Papers**								
			**ASD IS–Lab IS**		**ASD CP–ASD IS**		**ASD CP–Lab IS**	
Original data		MAD	0.005		0.009		0.011	
		MStD	0.007		0.009		0.010	
After lin. regression		MAD	0.003		0.004		0.005	
		MStD	0.004		0.006		0.007	
**NDI Differences: Tobacco**						**NDI Differences: Norway Spruce**		
		**ASD CP–ASD IS**	**ASD CP–Lab IS**	**ASD IS–Lab IS**				**ASD CP–ASD IS**
Original data	MAD	0.027	0.026	0.017		Original data	MAD	0.062
	MStD	0.026	0.028	0.019			MStD	0.029
After lin. regression	MAD	0.012	0.015	0.014		After lin. regression	MAD	0.019
	MStD	0.016	0.021	0.019			MStD	0.024

**Table 8:** Content of the biochemical parameters in the collected samples—Chlorophyll a/b (Cab), Carotenoids (Car) and relative water content (RWC). The samples for ASD CP measurements were taken from slightly different parts of the leaves than samples for the ASD IS and Lab IS (for a detailed explanation see Figure 1). CP—Contact probe, IS—Integration sphere, ASD, Lab—Producers of the measurement devices.

Parameter	Tobacco Leaves ASD CP		Tobacco Leaves ASD/Lab IS		Norway Spruce Needles	
	Mean	StD	Mean	StD	Mean	StD
Cab [μg/cm^2^]	21.3	5.0	20.5	4.8	38.9	11.0
Car [μg/cm^2^]	2.4	0.5	2.3	0.6	5.1	1.3
RWC [%]	84.7	5.9	85.3	5.8	57.9	2.5

**Table 9:** The coefficient of determination R^2^ showing the strength of the linear dependence between selected spectral indices derived from measured spectra and results of biochemical analyses for tobacco and Norway spruce samples. Only indices with the highest R^2^ value are presented. In parenthesis, mean value and standard deviation of spectral indices are given. Cab—Total chlorophyll, Car—Total carotenoids, RWC—Relative water content; for explanation of spectral indices see Table 4.

Tobacco				
Parameter	Spectral Index	R^2^ (Mean ± Stand Deviation)		
		ASD CP	ASD IS	Lab IS
Cab [μg/cm^2^]	OSAVI 2	0.63 (0.272 ± 0.069)	0.64 (0.281 ± 0.041)	0.65 (0.288 ± 0.052)
Car [μg/cm^2^]	R_NIR_CRI_550_	0.15 (1.87 ± 0.53)	0.21 (1.66 ± 0.20)	0.16 (1.64 ± 0.32)
RWC [%]	MSI	0.03 (0.602 ± 0.029)	0.15 (0.636 ± 0.026)	0.06 (0.631 ± 0.029)
**Norway Spruce**				
**Parameter**	**Spectral Index**	**ASD CP**	**ASD IS SE**	
Cab [μg/cm^2^]	TCARI	0.48 (0.171 ± 0.038)	0.46 (0.261 ± 0.061)	
Car [μg/cm^2^]	CRI700	0.20 (6.52 ± 1.69)	0.10 (3.99 ± 0.81)	
RWC [%]	NDWI	0.43 (0.120 ± 0.021)	0.07 (0.066 ± 0.011)	
